# Prevalence and Factors of Gallstone Disease in the High-Altitude Tribal Population of Spiti Valley, Northern India: A Population-Based Cross-Sectional Study

**DOI:** 10.7759/cureus.110729

**Published:** 2026-06-12

**Authors:** Vipan Kumar, Viper Sharma, Sushma Makhaik, Sandeep Rajta, Digvijay Thakur, Chaman Thakur, Sudhakar Chugh

**Affiliations:** 1 Surgery/Surgical Gastroenterology, Indira Gandhi Government Medical College & Hospital, Shimla, IND; 2 Surgical Gastroenterology, Postgraduate Institute of Medical Education and Research, Chandigarh, IND; 3 Surgery, Army Hospital Research and Referral, Delhi, IND; 4 Nuclear Medicine, Dr. Radhakrishnan Govt. Medical College, Hamirpur, Hamirpur, IND; 5 Radiology, Indira Gandhi Government Medical College & Hospital, Shimla, IND; 6 General Surgery, Indira Gandhi Government Medical College & Hospital, Shimla, IND; 7 Neurosurgery, Indira Gandhi Government Medical College & Hospital, Shimla, IND

**Keywords:** epidemiology, gallstone disease, high altitude, himalayan population, population-based study, spiti valley, ultrasonography

## Abstract

Background: Gallstone disease shows marked geographic variation, but epidemiological data from native Himalayan high-altitude populations remain limited. This study estimated gallstone prevalence in native adults from Spiti Valley and evaluated whether altitude was independently associated with disease after accounting for demographic and metabolic factors.

Methods: This community-based cross-sectional study was conducted in Spiti Valley, Himachal Pradesh, India. Using stratified random sampling, 450 native residents aged 30-70 years were enrolled across three altitude strata: low (<3500 m), mid (3501-4000 m), and high (>4000 m), with 150 participants in each group. All participants underwent fasting abdominal ultrasonography. Age, sex, body mass index, hypertension, diabetes mellitus, *Helicobacter pylori* status, fatty liver, and hepatitis B surface antigen status were recorded. Variables considered clinically relevant, together with the altitude group, were entered into multivariable logistic regression.

Results: Overall gallstone prevalence was 21.3% (96/450). Crude prevalence was 24.0% in the low-altitude group, 24.7% in the mid-altitude group, and 15.3% in the high-altitude group; the difference across strata was not statistically significant (p=0.089). Participants with gallstone disease were older and had higher body mass index and more frequent fatty liver on univariate analysis. In the multivariable model, age (aOR 1.04 per year, 95% CI 1.01-1.06; p=0.001), female sex (aOR 2.04, 95% CI 1.19-3.51; p=0.010), and fatty liver (aOR 1.79, 95% CI 1.05-3.06; p=0.032) remained independently associated, whereas altitude did not.

Conclusion: Native Spiti highlanders had a high overall gallstone burden, but the principal signal was not an independent altitude effect. This finding highlights a high-risk native Himalayan population.

## Introduction

Gallstone disease (GSD) is a common hepatobiliary disorder with substantial geographic, demographic, and metabolic variation worldwide. A recent meta-analysis estimated that gallstones affect approximately 6% of the global population, with a higher prevalence in women and in South America [1].

India likewise demonstrates marked regional variation in the burden of GSD. Earlier community-based studies from North India reported prevalence rates of 4.15% in the rural Gangetic basin and 6.12% in Kashmir [2,3].

A recent large, multicentric, community-based study from India, based on self-reported gallstone history, also demonstrated substantial regional variation, with prevalence ranging from 0.3% in Barshi to 10.7%-10.8% in Kamrup (Guwahati) and Mullanpur [4].

These observations indicate that the burden of GSD in India is regionally variable and may be influenced by demographic, environmental, and possibly lifestyle-related factors.

Data from high-altitude populations remain limited and conflicting. In a community-based ultrasonographic survey of three rural Peruvian villages located above 3000 m, Moro et al. reported age-adjusted gallstone prevalence rates of 4%-10% in men and 18%-20% in women. Prevalence increased significantly with age, whereas body mass index (BMI) showed a positive but non-significant association [5].

In contrast, their subsequent study comparing Peruvian coastal natives, highland migrants, and Andean villagers concluded that high altitude was not an independent positive risk factor for GSD and suggested that population or ethnic background may be more important than altitude alone [6].

More recent evidence from the Qinghai-Tibet Plateau has reopened this question by identifying altitude as an independent risk factor for GSD in a large employee health-check cohort. However, the authors acknowledged that their sample consisted of employees of formal institutions and therefore could not be directly generalized to the wider plateau population [7].

Population-based studies from Taiwan and other regions have identified age and metabolic correlates, including fatty liver, as important determinants of GSD, while community-based studies have consistently reported a higher burden in women. Taken together, these findings suggest that any apparent altitude-related association is likely to coexist with, or be confounded by, conventional demographic and metabolic determinants [7-9].

Against this background, epidemiological data on GSD in native Himalayan high-altitude populations remain scarce. The Spiti Valley of the Greater Himalayas, with its marked altitude gradient and relatively stable native population, provides an important setting for a community-based evaluation of GSD.

This population-based ultrasonographic study was therefore undertaken to estimate the prevalence of GSD across predefined altitude strata in native residents of Spiti Valley and to evaluate whether altitude was independently associated with GSD after accounting for selected demographic, clinical, and metabolic factors.

## Materials and methods

Study design and setting

This community-based, population-based cross-sectional study was conducted from February 2024 to January 2026 in the Spiti Valley of Himachal Pradesh, India. The primary objective was to estimate the prevalence of GSD among adult native highland residents of the Spiti Valley using community-based ultrasonographic screening. The secondary objective was to evaluate whether altitude and selected demographic, clinical, metabolic, and dietary/lifestyle factors were associated with GSD after adjustment for relevant covariates.

The study was designed and reported in accordance with the Strengthening the Reporting of Observational Studies in Epidemiology (STROBE) guidelines for cross-sectional observational studies.

Participants, sampling, and data collection

Permanent native residents of the study area were identified from the electoral roll and selected using stratified random sampling across three predefined altitude strata: low altitude (≤3500 m), mid-altitude (3501-4000 m), and high altitude (>4000 m).

The high-altitude stratum included residents of Hikkim (4440 m) and Komik (4587 m), among the highest permanently inhabited villages in the Spiti Valley that are accessible by a motorable road. Study villages within each altitude stratum were selected by randomization, and eligible individuals were then chosen using a computer-generated random sequence. To account for anticipated non-availability, non-response, and field-level exclusions, approximately 20% additional individuals were initially sampled.

Participants who met the inclusion and exclusion criteria and provided written informed consent were enrolled. Eligible individuals were aged 30-70 years, as the protocol restricted enrollment to this age group, given that GSD is less frequent at younger ages. The protocol permitted the inclusion of permanent native residents or second-generation native residents of Spiti Valley. The final study cohort comprised 450 participants, with 150 individuals in each altitude group. Individuals with prior cholecystectomy, incomplete essential data, or technically unreliable ultrasonographic assessment were excluded from the final prevalence analysis.

Data were collected using a structured proforma and included age, sex, height, weight, BMI, hypertension, diabetes mellitus, *Helicobacter pylori* status, and hepatitis B surface antigen (HBsAg) status.

All participants underwent abdominal ultrasonography after overnight fasting for the detection of GSD and other gallbladder-related findings, including fatty liver, gallbladder polyps, thick-walled gallbladder, and ultrasonographic suspicion of gallbladder malignancy. Ultrasonography was performed using a Toshiba Famio 5S ultrasound machine, with a standard curvilinear abdominal probe used for abdominal imaging, operating in the low-frequency abdominal range of approximately 3.5-5 MHz. The examinations were performed by two trained operators experienced in abdominal ultrasonography, including a radiology senior resident authorized to operate the machine during the field camp.

GSD was diagnosed by the presence of echogenic intraluminal foci within the gallbladder producing posterior acoustic shadowing, with or without mobility. Fatty liver was recorded using standard ultrasonographic features, including increased hepatic echogenicity relative to the renal cortex, vascular blurring, and posterior beam attenuation. Abdominal ultrasonography served as the primary modality for case ascertainment and prevalence estimation in this screening study.

Formal inter-observer reliability testing was not performed. Complete blinding of the operators to village or altitude category was not feasible in the field setting; however, the operators were not involved in statistical analysis.

Sample size

According to the study protocol, the minimum sample size for prevalence estimation was calculated using an expected GSD prevalence of 10%-15% in the general population, a 95% confidence level, and an absolute precision of 5%, yielding a minimum required sample size of 373 participants. To ensure balanced representation across the three predefined altitude strata and to allow altitude-wise comparisons, the planned enrollment was increased to 450 participants, with an equal allocation of 150 participants in each altitude group. This design provided adequate precision for estimating overall and stratum-specific prevalence and permitted comparison across altitude groups; however, the study was not specifically powered to detect small differences in gallstone prevalence between altitude strata.

Definitions

GSD was defined as the presence of gallstones on abdominal ultrasonography. The primary outcome was the prevalence of GSD in the study cohort overall and across the three predefined altitude strata. Fatty liver was recorded on ultrasonographic assessment. Hypertension, diabetes mellitus, and *Helicobacter pylori* positivity were analyzed as binary variables.

Statistical analysis

Continuous variables were expressed as mean ± standard deviation, and categorical variables as frequency and percentage. Comparisons across altitude strata were performed using one-way analysis of variance (ANOVA) for continuous variables and the chi-square test or Fisher’s exact test for categorical variables, as appropriate. Participants with and without GSD were compared using univariate analysis. Variables considered clinically relevant, along with the altitude group, were entered into a multivariable binary logistic regression model to identify factors independently associated with GSD. Adjusted odds ratios (aORs) with 95% confidence intervals (CIs) were calculated. A two-sided p-value of <0.05 was considered statistically significant. Because participants were recruited from villages within predefined altitude strata, the possibility of village-level clustering was considered. However, the primary analysis was performed at the individual-participant level, and formal cluster-adjusted or multilevel modeling was not performed because of the limited number of village clusters within some altitude strata. Participants with prior cholecystectomy, incomplete essential data, or technically unreliable ultrasonographic assessment were excluded from the final prevalence analysis. Complete-case data were used for the final analysis. Variables entered into the multivariable logistic regression model were selected a priori based on clinical relevance, biological plausibility, available literature, and altitude group as the exposure of interest, rather than solely on univariate statistical significance.

Ethical considerations

The study was approved by the Institutional Ethics Committee (IEC) of Indira Gandhi Medical College, Shimla, Himachal Pradesh, India (vide letter no. HFW(MC-II) B (12) ETHICS/2024/, dated 02 May 2024). Written informed consent was obtained from all participants prior to enrollment. Participant confidentiality was maintained throughout the study, and all analyses were performed on anonymized data.

## Results

A total of 450 participants were included in the analysis, with 150 participants each in the low-altitude (<3500 m above mean sea level), mid-altitude (3501-4000 m above mean sea level), and high-altitude (>4000 m above mean sea level) groups.

Baseline characteristics differed significantly across altitude strata (Table 1). The mean age was highest in the low-altitude group and lowest in the high-altitude group (48.5 ± 12.9 vs. 42.8 ± 12.0 years; p < 0.001).

**Table 1:** Baseline characteristics of the study cohort across altitude strata Continuous variables are presented as mean ± standard deviation and categorical variables as number (percentage). P-values were calculated using one-way analysis of variance for continuous variables and the chi-square test or Fisher’s exact test for categorical variables, as appropriate. Altitude is expressed above mean sea level.

Variable	Low altitude (<3500 m), n = 150	Mid altitude (3501–4000 m), n = 150	High altitude (>4000 m), n = 150	P-value
Age (years), mean ± SD	48.5 ± 12.9	44.5 ± 14.8	42.8 ± 12.0	<0.001
Female sex, n (%)	101 (67.3)	79 (52.7)	110 (73.3)	<0.001
BMI (kg/m²), mean ± SD	23.4 ± 4.3	23.9 ± 3.9	21.1 ± 3.8	<0.001
Fatty liver, n (%)	52 (34.7)	45 (30.0)	39 (26.0)	0.262
Hypertension, n (%)	12 (8.0)	10 (6.7)	3 (2.0)	0.059
Diabetes mellitus, n (%)	4 (2.7)	2 (1.3)	2 (1.3)	0.601
*H. pylori* positive, n (%)	77 (51.3)	87 (58.0)	100 (66.7)	0.026
HBsAg positive, n (%)	26 (17.3)	14 (9.3)	26 (17.3)	0.078

BMI also differed significantly across altitude strata, with a lower mean BMI in the high-altitude group (21.1 ± 3.8 kg/m²) than in the low-altitude (23.4 ± 4.3 kg/m²) and mid-altitude (23.9 ± 3.9 kg/m²) groups (p < 0.001).

The proportion of women also varied significantly across altitude groups, with the highest in the high-altitude group (73.3%) and the lowest in the mid-altitude group (52.7%) (p < 0.001). *Helicobacter pylori* positivity likewise differed significantly across groups, ranging from 51.3% in the low-altitude group to 66.7% in the high-altitude group (p = 0.026).

The overall prevalence of GSD in the study cohort was 21.3% (96/450). The prevalence was 24.0% (36/150) in the low-altitude group, 24.7% (37/150) in the mid-altitude group, and 15.3% (23/150) in the high-altitude group (Table 2).

**Table 2:** Crude prevalence of gallstone disease across altitude strata Prevalence is presented as a percentage with a 95% confidence interval. Overall comparison across altitude strata by chi-square test: p = 0.089. Altitude is expressed above mean sea level.

Altitude group	Total, n	GSD cases, n	Prevalence, % (95% CI)
Low altitude (<3500 m)	150	36	24.0% (17.4–31.6)
Mid-altitude (3501–4000 m)	150	37	24.7% (18.0–32.4)
High altitude (>4000 m)	150	23	15.3% (10.0–22.1)
Overall	450	96	21.3% (17.6–25.4)

Although the high-altitude group had the lowest crude prevalence, the overall difference across altitude strata did not reach statistical significance (p = 0.089). When participants with and without GSD were compared, those with GSD were significantly older (51.0 ± 12.7 vs. 43.7 ± 13.2 years; p < 0.001) and had a higher BMI (24.1 ± 5.3 vs. 22.4 ± 3.8 kg/m²; p = 0.006) (Table 3).

**Table 3:** Comparison of participants with and without gallstone disease Continuous variables are presented as mean ± standard deviation and categorical variables as number (percentage). P-values were calculated using the independent-samples t test for continuous variables and the chi-square test or Fisher’s exact test for categorical variables, as appropriate. Interpretation of diabetes mellitus should be cautious because of the low absolute number of diabetic participants.

Variable	No gallstone disease (n = 354)	Gallstone disease (n = 96)	P-value
Age (years), mean ± SD	43.7 ± 13.2	51.0 ± 12.7	<0.001
Female sex, n (%)	220 (62.1)	70 (72.9)	0.067
BMI (kg/m²), mean ± SD	22.4 ± 3.8	24.1 ± 5.3	0.006
Fatty liver, n (%)	95 (26.8)	41 (42.7)	0.004
*H. pylori* positive, n (%)	217 (61.3)	47 (49.0)	0.039
Diabetes mellitus, n (%)	2 (0.6)	6 (6.2)	0.002
Hypertension, n (%)	18 (5.1)	7 (7.3)	0.558
HBsAg positive, n (%)	48 (13.6)	18 (18.8)	0.266

Females showed a non-significant trend toward higher frequency among participants with GSD (72.9% vs. 62.1%; p = 0.067). Fatty liver was significantly more common in participants with GSD (42.7% vs. 26.8%; p = 0.004). In contrast, *Helicobacter pylori* positivity was less frequent among participants with GSD than among those without GSD (49.0% vs. 61.3%; p = 0.039). Diabetes mellitus was also more frequent in the GSD group; however, the absolute number of diabetic participants was very small, and this finding should therefore be interpreted cautiously.

On multivariable logistic regression analysis adjusting for altitude group, age, BMI, sex, *Helicobacter pylori* status, fatty liver, HBsAg status, and hypertension, age, female sex, and fatty liver remained independently associated with GSD (Table 4).

**Table 4:** Multivariable logistic regression analysis of factors independently associated with gallstone disease Low altitude was used as the reference category for altitude. Male sex, absence of *Helicobacter pylori* positivity, absence of fatty liver, absence of HBsAg positivity, and absence of hypertension were used as reference categories. Adjusted odds ratios were derived from a multivariable binary logistic regression model. BMI, body mass index; HBsAg, hepatitis B surface antigen.

Variable	Adjusted odds ratio	95% confidence interval	P-value
Mid vs. low altitude	1.35	0.76 to 2.38	0.306
High vs. low altitude	0.78	0.42 to 1.47	0.446
Age (per year)	1.04	1.01 to 1.06	0.001
BMI (per kg/m²)	1.04	0.98 to 1.11	0.219
Female sex	2.04	1.19 to 3.51	0.010
H. pylori positive	0.75	0.46 to 1.24	0.265
Fatty liver	1.79	1.05 to 3.06	0.032
HBsAg positive	1.32	0.69 to 2.51	0.406
Hypertension	0.76	0.28 to 2.05	0.590

Each one-year increase in age was associated with a 4% increase in the odds of GSD (aOR 1.04, 95% CI 1.01-1.06; p = 0.001). Female participants had approximately two-fold higher odds of GSD than male participants (aOR 2.04, 95% CI 1.19-3.51; p = 0.010). Fatty liver was also independently associated with GSD (aOR 1.79, 95% CI 1.05-3.06; p = 0.032). In contrast, altitude group, BMI, *Helicobacter pylori* positivity, HBsAg positivity, and hypertension were not independently associated with GSD.

All 450 participants underwent abdominal ultrasonography. Other ultrasonographic gallbladder abnormalities were uncommon, including gallbladder polyps in five participants (1.1%), thick-walled gallbladder in six (1.3%), and ultrasonographic suspicion of gallbladder malignancy in four (0.9%); detailed distribution is provided in Table 5. Because of the very small numbers, these findings were not included in the primary comparative or multivariable analyses.

**Table 5:** Other ultrasonographic gallbladder abnormalities detected in the study cohort Values are presented as numbers (percentages). Percentages are based on 150 participants in each altitude stratum and 450 participants overall. Because of the small number of events, no inferential comparison was performed.

Finding	Low altitude (<3500 m)	Mid-altitude (3501–4000 m)	High altitude (>4000 m)	Overall
Gallbladder polyp, n (%)	2 (1.3)	2 (1.3)	1 (0.7)	5 (1.1)
Thick-walled gallbladder, n (%)	0 (0.0)	6 (4.0)	0 (0.0)	6 (1.3)
USG suspicion of gallbladder malignancy, n (%)	0 (0.0)	4 (2.7)	0 (0.0)	4 (0.9)

## Discussion

In this population-based ultrasonographic study of native residents of the Spiti Valley, the overall prevalence of GSD in the study cohort was 21.3%, with crude prevalence rates of 24.0% in the low-altitude group, 24.7% in the mid-altitude group, and 15.3% in the high-altitude group.

Although the high-altitude group had the lowest crude prevalence, altitude was not independently associated with GSD after multivariable adjustment. Instead, increasing age, female sex, and fatty liver emerged as the principal correlates of GSD in this cohort. These findings suggest that, in this Himalayan population, conventional demographic and metabolic factors are more important than altitude alone in explaining gallstone burden. The prevalence observed in our study cohort was substantially higher than that reported in most previous Indian community-based studies.

Earlier sonographic surveys from North India reported prevalence rates of 4.15% in the rural Gangetic basin [2] and 6.12% in Kashmir [3], while a recent large multicentric Indian community-based study based on self-reported gallstone history showed marked regional variation, ranging from 0.3% in Barshi to 10.7%-10.8% in Kamrup (Guwahati) and Mullanpur [4].

The higher prevalence observed in this cohort may reflect the distinctive demographic, environmental, dietary, and metabolic profile of this native Himalayan population, as well as the use of direct ultrasonographic screening in all participants rather than reliance on self-reported disease.

In Spiti Valley, extreme winter conditions, including temperatures below −30°C, may lead to prolonged indoor confinement and seasonal dependence on stored meat. Although these exposures were not formally measured in the present study, they may plausibly contribute to the local gallstone risk profile.

The question of whether altitude itself influences gallstone formation remains unresolved. Moro et al. reported that GSD was common in high-altitude rural Peruvian populations, with age-adjusted prevalence ranging from 4% to 10% in men and 18% to 20% in women. They also found that prevalence increased with age, whereas BMI showed only a positive but non-significant association [5].

However, in their subsequent Peruvian migration study, the same group found that age-adjusted prevalence did not differ significantly between coastal natives, highland migrants, and Andean villagers and concluded that high altitude was not an independent positive risk factor for GSD [6].

In contrast, a recent large study from the Qinghai-Tibet Plateau identified altitude as an independent risk factor for GSD in an employee health-check cohort. However, the authors also acknowledged that their sample was restricted to employees of formal institutions and could not be generalized to nomadic, farming, and other non-formally employed populations of the plateau [7].

Our findings are therefore more closely aligned with the Peruvian data than with the Qinghai study, in that altitude showed a crude difference across groups but did not remain independently associated with GSD after adjustment.

Age was independently associated with GSD in the present study, with the odds increasing progressively with advancing age. This finding is biologically plausible and consistent with population-based data from Taiwan and South-East Iran, both of which identified increasing age as a significant correlate of GSD [8,9].

The population-based study from Taiwan is particularly relevant, as it identified both age and fatty liver as important correlates of GSD in both sexes and highlighted fatty liver as a clinically meaningful metabolic factor in gallstone epidemiology [8].

The association with advancing age likely reflects cumulative exposure to lithogenic influences, including changes in bile composition, gallbladder motility, and long-term metabolic burden.

Female sex was independently associated with GSD in the present study, with women having approximately two-fold higher adjusted odds than men. This finding is consistent with previous epidemiological studies reporting a higher burden of GSD among women [10].

These findings support the continued importance of female biological and reproductive factors in gallstone epidemiology across diverse populations [11,12].

Fatty liver remained independently associated with GSD in the adjusted model, suggesting shared metabolic pathways between gallstone formation and hepatic steatosis. In contrast, BMI was higher among participants with GSD on univariate analysis but lost significance after adjustment, suggesting that generalized adiposity may be less informative than metabolic manifestations such as fatty liver.

The inverse association between *Helicobacter pylori* positivity and GSD in univariate analysis did not persist after adjustment and should therefore be interpreted with caution, particularly because the literature on this association remains inconsistent and methodologically heterogeneous [13-15].

An important strength of this study is its population-based design in a geographically distinctive Himalayan setting. Unlike hospital-based or symptom-driven series, this study used community screening with abdominal ultrasonography in all enrolled participants, allowing detection of both asymptomatic and symptomatic gallstones.

The equal inclusion of 150 participants in each altitude stratum permitted direct comparison across altitude groups. In addition, the study provides data from native residents of Spiti Valley, a population that remains underrepresented in the gallstone epidemiology literature.

Limitations

This study has several limitations. First, the cross-sectional design precludes causal inference; therefore, the observed associations should be interpreted as correlates of GSD rather than definitive causal risk factors. Although stratified sampling was used to ensure representation across altitude strata, selection and non-response bias cannot be completely excluded, as some initially sampled individuals were unavailable, declined to participate, or were excluded due to prior cholecystectomy, incomplete essential data, or technically unreliable ultrasonographic assessment. In addition, potential village-level clustering was not formally modeled, and participants from the same village may have shared dietary, environmental, socioeconomic, and lifestyle exposures.

Second, although all participants underwent ultrasonographic screening using standard diagnostic criteria, a formal interobserver reliability assessment was not performed, and complete blinding of ultrasound operators to village or altitude category was not feasible in the field setting. Third, the altitude groups differed significantly in baseline characteristics, including age, sex distribution, BMI, and *Helicobacter pylori* positivity. Although multivariable adjustment was performed, residual confounding remains possible. Important covariates, including detailed dietary intake, parity, reproductive history, family history, lipid profile, socioeconomic status, and physical activity, were not included in the final analysis. The small number of participants with diabetes mellitus and uncommon gallbladder abnormalities also limited the interpretation of these findings. Finally, ultrasonographic assessment of fatty liver, while appropriate for community screening, is operator-dependent and may not fully capture the severity or spectrum of steatotic liver disease [16,17].

## Conclusions

In this population-based ultrasonographic study of native residents of Spiti Valley, the study-cohort prevalence of GSD was high at 21.3%, exceeding many previously reported estimates from general population studies in India. Although crude prevalence varied across altitude strata, the difference was not statistically significant, and altitude was not independently associated with GSD after multivariable adjustment. Increasing age, female sex, and fatty liver were independently associated with GSD, suggesting that conventional demographic and metabolic factors may be more important than altitude alone in explaining the gallstone burden in this population. Because of the cross-sectional design, these findings should be interpreted as associations rather than causal risk factors.

The key novel finding of this study is the demonstration of a high gallstone burden in a native trans-Himalayan population without evidence that altitude itself is an independent driver. Further longitudinal studies incorporating detailed dietary, reproductive, genetic, lifestyle, and metabolic data are needed to clarify the basis of this elevated burden in Himalayan highland communities.
